# Psychological dynamics of overqualification: career anxiety and decision commitment in STEM

**DOI:** 10.1186/s40359-024-02061-5

**Published:** 2024-11-22

**Authors:** Ali Nawaz Khan, Mohsin Ali Soomro, Naseer Abbas Khan, Ali Ahmad Bodla

**Affiliations:** 1 Research Center of Hubei Micro & Small Enterprises Development, School of Economics and Management, Hubei Engineering University, Xiaogan, 432100 PR China; 2 Noon Business School, University of Sargodha, Sargodha, Pakistan; 3 Institute for Management Research, Radboud University, Nijmegen, 6525AJ the Netherlands

**Keywords:** Overqualification, Women in STEM, Career Identity, Career Decidedness, Leadership Support, Career anxiety

## Abstract

Women’s career progression and empowerment in STEM (science, technology, engineering, and mathematics) sectors are critical to attaining SDG 5: Gender Equality because they promote equal access to education, job prospects, and leadership roles, building a more inclusive and equitable society. The purpose of this study is to look into the impact of perceived overqualification (POQ) on career anxiety and career decidedness (CD) among women in STEM disciplines while also considering the function of career identity (CI) and leadership support. With a total sample size of 1,045 participants, two distinct investigations were conducted, one in the educational field (*N* = 530) and one in an industry setting (*N* = 515 time-lag). To test the model, the analysis was carried out using the AMOS-24 software program. Our findings show a favorable association between women’s perceptions of overqualification in STEM and their CI. Furthermore, our research shows that a stronger CI among women in STEM corresponds to decreased career anxiety and increased CD. Additionally, we find that a CI is a mediator between POQ and both career anxiety and CD. Our findings also highlight the moderating effect of leadership support in this mediation process. We discuss the theoretical and practical ramifications of these findings.

## Introduction

How can career identity (CI) and leader support turn potentially bad situations into opportunities for women in STEM fields who feel overqualified? Women’s advancement in STEM professions is extremely important in the pursuit of the UN Sustainable Development Goal 5(SDG5): Gender Equality. It not only assures equitable access to education, career prospects, and leadership positions, but it also lays the groundwork for a vibrant and welcoming society in which various voices can determine the future of innovation. Accepting the transformative impact of women’s STEM jobs is more than a goal; it is a catalyst for attaining SDG 5: Gender Equality. We create an open environment where their unique ideas and abilities can thrive by breaking down obstacles supporting diversity, and empowering women in STEM areas, pushing progress toward a fairer and thriving world. Women in STEM professions frequently have specific challenges such as problems with stereotype risk, gender bias, and discrimination. Feelings of frustration, work dissatisfaction, and limited chances for career progression can result from perceived overqualification, a situation in which women have talents and qualifications that surpass the criteria of STEM employment. With technological and innovation advancements and the global economy’s development, STEM employees are becoming increasingly important to the economic growth of countries. However, an important challenge is that far too many early careers STEM specialists leave the field [1, 2], in some cases because they feel overqualified [3]. Perceived overqualification (POQ) occurs when people think that their credentials exceed the requirements of their job [4]. Studies have shown that STEM professionals often feel overqualified for their current jobs and may consider pursuing careers in other sectors, with lower pay [5].

This research for women in STEM fields is critical as the demand for STEM professionals continues to increase in advanced economies. For example, there is a shortage of STEM practitioners in the United States [6–8]. However, many STEM professionals believe that they will not be well paid in the STEM sector despite their qualifications, due to the widespread stagnation in career advancement [9], and that now is the best time to switch to another profession, as lucrative jobs may become scarcer soon. Overqualification has been defined as a distinct form of human capital with distinct requirements, attracting attention from human resource (HR) management scholars [10]. Academics have emphasized the importance of improving HR practices, especially in STEM fields, as initiatives such as employee retention plans can help organizations leverage overqualified staff to meet organizational requirements while also meeting the specific needs of these employees [11]. It is critical to comprehend the unique requirements of the overqualified STEM workforce to develop corresponding HR policies. To this end, this study investigates the outcomes of overqualification for STEM specialists’ women and the needs of overqualified STEM specialists. According to [12], overqualification is associated with negative consequences. However, recent investigations have shown that overqualification can contribute to positive outcomes among employees, such as high levels of engagement [13] and engagement in extra-role activities [14].

The literature suggests that rather than considering POQ as intrinsically positive or negative, it is essential to investigate the circumstances that lead to favorable or unfavorable outcomes from overqualification. In this study, we attempt to reconcile the mixed findings of previous work by treating CI as a mediator and leader support as a moderator of the link between POQ and female employee outcomes in STEM fields. Role identity theory (RIT) posits that a person’s self-perception shapes his or her identity in a specific social role [4]. CI motivates and guides individuals to consciously adjust their circumstances to pursue their career interests [1, 15]. While scholars have demonstrated the value of role identity, we hypothesize that a strong CI is related to STEM employees’ POQ. Specifically, we expect STEM workers’ POQ to strengthen their career identity, in turn increasing their career decidedness (CD) and reducing their career anxiety (CA).

**Fig. 1:**
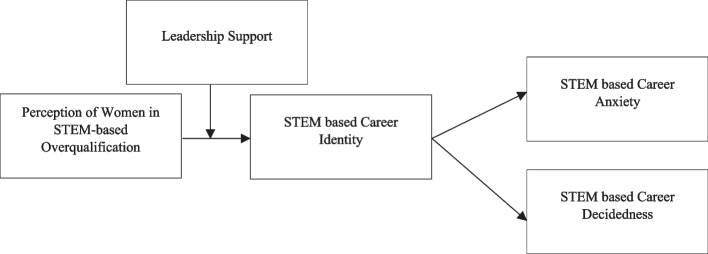
Research model

We also investigate whether leader support strengthens the link between employees’ POQ and CI, using the symbolic interactionist outlook of role identity [16, 17], which shows that positive social experiences help establish and sustain a positive role identity. We focus on leader support rather than other leadership behaviors because leader support (LS) is closely linked with RIT. Specifically, unqualified workers who are willing to show their ability to act and maintain an expression of dominance are more likely to have their successes highlighted by supportive leaders [18]. Social interaction in the workplace helps shape and sustain a person’s professional identity [19]. We contend that overqualified early-career female STEM professionals can participate in more constructive social interactions. Thus, when they are supported by their supervisors, workers in STEM-related jobs are better able to maintain or even strengthen their professional identity. The relationship shown in Fig. 1 is demonstrated in this analysis.

Current research makes significant contributions to the literature on female STEM overqualification, with an emphasis on HR management, leadership, and career behavior. Although research has recently begun to investigate how workers who feel overqualified can put their skills to good use [20], to the best of our knowledge, little evidence has been obtained of whether female overqualification has positive effects on STEM employees’ career outcomes. Furthermore, there has been limited research on STEM women’s overqualification in relation to their CD. Based on the idea that STEM overqualification reflects a mismatch between a STEM employee and his or her job [21], it is logical to assume that alleviating this mismatch will influence women’s potential career decisions [22].

This study aims to fill several gaps in the literature, first, unlike previous studies, we investigate female employee’s CA and CD as two distinct outcomes of STEM women’s POQ. Our study therefore fills a gap in the literature on work behavior and STEM overqualification by contrasting employees’ POQ with their career outcomes. Second, this study contributes to the HR management and organizational behavior literature by defining the career-related needs of early-career STEM professionals who think overqualified for their jobs. Our findings can help companies and HR practitioners develop viable training and development (T&D) strategies to empower early-career STEM professionals who feel overqualified to make better career decisions.

For various reasons, addressing the effect of perceived overqualification on women in STEM occupations is critical. Firstly, recognizing this phenomenon enables women to reframe overqualification as an asset rather than a burden. Women may construct a strong job identity, boost self-confidence, and navigate their STEM careers with resilience by understanding the potential negative impacts and developing remedies. This, in turn, aids their professional development and success. Second, women’s overqualification in STEM-related fields indicates an untapped reservoir of potential and skill. Organizations may establish cultures that use and optimize the abilities, expertise, and potential of women professionals by addressing the problems associated with perceived overqualification. This benefits not only individual women, but also the overall innovation, productivity, and competitiveness of STEM companies. Third, strong leadership support is critical for assisting women in transforming perceived overqualification issues into opportunities. Mentoring, counseling, and sponsorship schemes can be provided by organizations to support leadership development and the progress of overqualified women in STEM professions. This assistance helps to break down barriers, promote inclusion, and create a more diverse and equitable STEM workforce. Finally, tackling perceived overqualification aids in closing the gender gap in STEM professions. Organizations can promote gender equality and create a more inclusive and varied work environment by recognizing and resolving the prejudices and barriers that women face because of overqualification.

## Conceptual background and hypotheses

### STEM-based POQ and STEM-based CI

The research on overqualification has typically concentrated on justice theories and person-environment fit theory [23]. Studies have employed justice theories to elucidate the behavioral and psychological responses of overqualified employees compared to their peers. In this study, we use [17] role identity model as our key theoretical model because it complements our temporal emphasis on the dependent variables. Unlike attitudes and behaviors in the workplace, employees’ career attitudes and behaviors reflect their long-term goals [24]. We use RIT to examine how POQ influences STEM employees’ career outcomes via their CI, provided that their personal identity system is relatively resilient and stable over time [25].

People’s various roles in society give them a sense of self and meaning [17, 26]. A person’s view of himself or herself in a specific role is referred to as role identification [27, 28]. The two key sources of role identity are (a) social feedback and (b) allied self-perceptions [29]. Role identity is an essential motivation for personal actions; specifically, it inspires people to act in ways that are compatible with their roles [30]. Individuals describe themselves in relation to their careers based on their decisions to pursue different career paths throughout their lives. CI may be seen as a key form of career identity that motivates workers to achieve their career goals.

We assume that STEM employees perceived overqualification strengthens their career identity, based on RIT. An individual’s role identity is shaped by his or her understanding of himself or herself and his or her interactions with others. Overqualified employees often believe that they are fantastic at their jobs [29] and more positive about themselves than about their coworkers. According to [18], individuals who experience overqualification have a positive self-perception. We expect staff who feel overqualified to set more ambitious career goals and to have more confidence in their ability to achieve these goals, given the connection between POQ and positive self-efficacy.

According to RIT, possessing a positive self-image in a specific role contributes to the formation of a more robust identity linked with that role [30]. We argue that the positive self-perception of overqualified STEM employees early in their careers helps them develop a strong STEM CI. The prerequisite for role identification is social interactions at work, which can lead to success in a specific role. Those who perceive themselves as overqualified can leverage their advanced skills and knowledge to maintain positive relationships with supervisors and colleagues [13]. Research has shown that POQ prompts employees to expand the scope of their work, learn new skills [29], and become more efficient in performing work-related tasks [13]. Therefore, we argue that POQ encourages STEM employees to act in ways that foster positive social relations with their subordinates and peers. According to previous research, colleagues who believe overqualified early career employees are more likely to seek support and advice from their peers through mentoring programs [20]. As positive social interactions are crucial for developing a strong role identity, we hypothesize that people who feel overqualified in STEM fields have a strong career identity [4]. Thus, we propose the following:

- H1: Perception of STEM-based overqualification and STEM-based career identity are positively related to each other.

### STEM-based CI and STEM-based CA

Anxiety is often interpreted as an emotional state or a personality trait. According to [31], CA is characterized by a propensity to react to a threatening situation. Generalized career anxiety disorder is based on the characteristics of employees in the workplace and refers to their tendency to respond to a wide range of career conditions with different levels of anxiety. CA has been attributed to a weak career identity. According to [32], an underdeveloped career identity can contribute to employees’ career anxiety. After unsuccessful career attempts and the subsequent loss of career identity, employees experience higher levels of CA [33–35]. CI has also been shown to be linked to self-perceived employability [36], which informs our understanding of CA. Therefore, in this study, we hypothesize that having a stronger (weaker) CI reduces (increases) career anxiety. Individuals with a strong sense of self are more likely to act in accordance with their positions [17]. We expect early-career STEM workers with a strong career identity to be able to participate in activities that help them advance their careers while reducing their CA.

Early-career STEM professionals who exhibit a high level of expertise are confident in their capacity to secure employment in their desired fields and to navigate their careers corresponding to their preferences. As a result, early-career STEM workers with a strong career identity are less likely to be uncertain about their career decisions and more likely to be proactive in gathering useful information, thereby reducing their career anxiety [37]. According to role identity theory, a strong career identity also provides workers with valuable emotional capital [4], as identity has both cognitive and emotional components. For example, employees with a robust CI tend to view themselves as significant individuals. They typically have an optimistic outlook regarding their employment opportunities and are less likely to worry about work-related issues [33]. When they face career setbacks, these individuals are likely to be resistant and engage in effective anxiety management. As a result, we expect early-career STEM workers with a stronger career identity to have less career anxiety. Therefore, we propose the following hypothesis:

- H2a: STEM-based career identity and STEM-based career anxiety have a negative relationship.

Furthermore, we argue that STEM-based CI mediates the effect of STEM-based POQ on STEM-based career anxiety. Specifically, we suggest that the career anxiety of overqualified STEM employees is reduced by their CI. Based on RIT we extrapolate that STEM-based POQ foster STEM based CI because employees’ positive perception of themselves makes them work vibrantly (H1). In turn, the STEM based career identity helps in mitigating or countering job anxiety of overqualified employees as they are likely to feel secure about their job and in control of their career progress with greater prospects (H2a). Hence, we speculate STEM based overqualification indirectly affects the job anxiety of STEM based overqualified employee through stronger STEM based CI. Thus, we formulate hypothesis H2b as below.

- H2b: STEM-based CI mediates the link between POQ and STEM-based employee CA.

### STEM-based career identity and STEM-based career decidedness

Career decidedness is defined as an individual’s level of confidence or certainty in their intended career path [38]. Over the last three decades, many career psychology scholars have tried to identify and define professional indecision. However, according to [39], no studies have investigated the psychological dimensions of career indecision and CI. Individuals use their actions to confirm and restore their career identity. As a result, an individual’s role identity can be seen as a motivator to behave in a particular way [30, 40]. According to role identity theory, individuals with a strong career identity typically monitor and reconstruct their identity in accordance with their roles [41]. According to [4], workers with a stronger CI are more likely to make decisions and actively initiate changes in the workplace. We argue that having a strong career identity helps people to make better career decisions by giving them a strong sense of purpose and future direction. Individuals with a strong CI are likely to have straightforward personal goals and aspirations, which facilitate their career decisions. This argument is supported by Fugate, Kinicki, and [4] in their study of occupational identity and employability. They show that a strong CI can predict career determinism, which is expressed through constructive initiatives such as defining potential career growth and adjusting the work environment to meet career needs.

In contrast, people with a weak career identity are unable to set career goals [42]. Early career employees are likely to change jobs if they are overqualified for their current roles [43]. When combined, these factors make career decisions difficult for early-career employees who have a weak career identity [42]. Thus, we expect early-career STEM workers with a strong career identity to make more career decisions than employees with a weak CI. Therefore, we propose the following hypothesis:

- H3a: STEM-based CI and STEM-based CD have a positive relationship.

We also hypothesize that early-career STEM employees who feel overqualified are more involved in CD and that their CI mediates this relationship. As mentioned above, POQ can be understood as a mismatch between people and their jobs [43]. Individuals who feel overqualified are expected to make CD to address the mismatch between their skills and their current jobs. Therefore, building on RIT we expect that STEM based POQ strengthen the STEM based career identity in early career-career individuals as they perceive themselves overqualified and take part in their jobs conveniently thus developing STEM based career identity (H1). Ensuing to their STEM based CI is their STEM based CD, because being convenient at their job, they take part in career decision making to match their skills and job (H3a). Therefore, we anticipate that STEM based overqualification will foster STEM based career decidedness through STEM based career identity in early-career individuals. Thus, we note H3b as below. Hence, we propose the following hypothesis:

- H3b: STEM-based CI mediates the link between POQ and CD.

### Moderating effects of Leadership support (LS)

According to [44], positive social interactions at work help workers understand their worth and develop a positive self-perception in the workplace, reflecting the symbolic interactionist perspective on CI. The existing research [30] argued that individuals’ interactions with their colleagues help them build and maintain their role identity. Leadership influences the social roles of workers and has been widely regarded as one of the most influential contextual variables [45]. In the current research, we argue that leader support may be an important moderator of the relationship between POQ and CI. We hypothesize that POQ by early career staff is more attributable to a stronger CI when leaders provide motivation. When leaders understand and respect the skills of their subordinates [46], they give them more chances to use these skills in the workplace.

Employees with higher qualifications tend to cultivate a robust self-identity and elevated professional aspirations when guided by supportive leaders. However, some leaders fail to support their employees, providing criticism rather than encouragement [36]. The subordinates of these leaders may assume that they are incompetent, undervalued, or underestimated. Unsupportive leaders are often more inclined to take risks that erode the self-efficacy of overqualified employees, causing them to doubt themselves [9]. Subordinates might struggle to establish a solid self-concept regarding their career objectives and aspirations if they rely on feedback from unsupportive supervisors to shape their self-perception. Thus, we hypothesize that if their leaders are more supportive, early-career STEM employees who feel overqualified will have a stronger CI.

- H4: LS moderates the positive interaction between the perception of STEM-based overqualification and STEM-based CI, so that when LS is high, the association becomes stronger, and vice versa.

We propose that early career STEM workers who feel overqualified are highly skilled and have a strong career identity, minimizing their career anxiety and enhancing their professional decision-making abilities. The contribution of CI to the positive link between POQ and career attitudes and behaviors is greater if employees’ leaders accept and respect them. Inadequate leader support weakens the relationship between POQ and CI, reducing indirect effects. Therefore, we propose the following hypotheses:

- H5a: LS moderates the indirect link between STEM-based POQ and employee STEM-based CA through STEM-based CI, making this indirect relationship stronger when LS is high versus low and weaker when LS is low.
- H5b: LS moderates the indirect link between STEM-based POQ and employee STEM-CA through STEM-based CI, making this indirect relationship stronger when LS is high and weaker when LS is low.

## Methods

### Data collection and procedures

We collected data from 530 early-career STEM female employees at 25 educational institutions (excluding universities) in China to test H1–H5b. In Study 2, we used time-lag data from 515 small and medium-sized engineering firms’ employees to replicate the findings of Study 1 and examine H4–H5b. Informed consent was obtained from all female employees, and the ethical guidelines provided by the educational institutions and the HR departments of the engineering firms were followed strictly. In both studies, we used a questionnaire with closed-ended questions based on items from existing scales, modified to match our context in terms of wording, and used a 5-point Likert scale.

#### Study 1

We distributed the questionnaires in Chinese to female faculty members (from STEM departments) at 25 educational institutions with the help of the administrative staff of the relevant departments. They completed the questionnaire during their progress review meeting in about 30 min. We received 530 usable responses from 860 respondents, for a 62% response rate. As shown in Table 1, most of the participants were having a mean age of 24.62 years, and most of them had obtained a master’s degree or a Ph.D. Moreover, the mean experience was 4.53 years.

**Table 1:** Study 1 & 2 descriptive statistics

Constructs	Mean	SD	1	2	3		4		5		6	7	8
**1. Age** ^**1**^	24.62	3.36	-										
**2. Experience**^**1**^	4.53	2.55	0.06	-									
**3. Education**	2.12	0.75	0.63^***^	0.02	-								
**4. Overqualification**	3.19	1.15	0.04	− 0.08	− 0.01		**(0.92)**						
**5. Career Identity**	2.86	1.10	− 0.01	0.03	− 0.03		0.24^**^		**(0.80)**				
**6. STEM Career Decidedness**	3.27	1.22	0.04	− 0.01	0.04		0.39^***^		0.48^***^		**(0.87)**		
**7. STEM Career Anxiety**	3.11	1.01	0.03	− 0.06	0.01		− 0.30^***^		− 0.28^**^		− 0.14^*^	**(0.85)**	
**Study 2**													
**1. Age** ^**1**^**(T1)**	25.30	3.79	-										
**2. Experience**^**1**^**(T1)**	5.72	3.96	0.14^*^	-									
**3. Education (T1)**	1.96	0.86	0.62^***^	− 0.04	-								
**4. Overqualification (T1)**	3.06	1.27	− 0.07	− 0.03	− 0.06	**(0.93)**							
**5. Career Identity (T2)**	2.71	1.14	− 0.01	0.04	− 0.07	0.33^***^		**(0.81)**					
**6. Leader Support (T2)**	3.05	1.14	− 0.06	− 0.01	− 0.09	0.06		0.21^**^		**(0.77)**			
**7. STEM Career Decidedness (T3)**	3.01	1.25	− 0.04	− 0.02	− 0.05	0.50^***^		0.44^***^		0.15^*^		**(0.85)**	
**8. STEM Career Anxiety (T3)**	3.06	1.11	0.06	0.05	0.10^*^	− 0.45^***^		− 0.33^***^		− 0.05		− 0.26^**^	**(0.86)**

(1) Age and experience are in years

Study 1: *N* = 530. Study 2: *N* = 515

α-values are indicated in parentheses

*T1* Time 1, *T2* Time 2, *T3* Time 3

*p < .05

**p < .01

***p < .001

### Measures

#### STEM-based POQ

In Study 1, following [47], we employed the 9-item POQ scale to measure STEM employees’ POQ, developed by [48]. Item included “*My job in a STEM field requires less education than I have*.”

#### STEM-based CI

To measure the respondents’ CI, we used the 4-item CI scale, developed by [49]. Item included “*I know who I am professionally in my STEM career.*”

#### STEM-based CA

To measure the respondents’ levels of CA in Study 1, we adopted the 5-item CA scale proposed by [50]. Item included “*I am worried about a future STEM career because my salary will probably not be as high as I wish.*”

#### STEM-based CD

To measure the CD, we used the 5-item scale, developed by [51]. This scale was used with employees and students by [51]. Item included “*I can list alternatives for my career.*”

## Data analysis

To validate our measurements, the discriminant validity of all the constructs was assessed by conducting confirmatory factor analysis (CFA). The CFA results for the four-factor model (POQ, CI, CA, and CD) showed that the fit indices for the proposed model were within acceptable ranges compared with other models (chi-square to degrees of freedom ratio [χ^2^/df] = 2.11, root mean square error of approximation [RMSEA] = 0.05, Tucker–Lewis index [TLI] = 0.95, comparative fit index [CFI] = 0.96), indicating that common method bias (CMB) was not a serious issue in our research, signifying that there were no reliability issues.

**Table 2:** Study 1 & 2 CFA results

**Models**	**χ2 (df)**	**χ2**_**diff**_**(df**_**diff**_**)**	**χ2/df**	**TLI **	**CFI **	**RMSEA**
**Four Factors Model**	473 (224)	-	2.11	.95	.96	.05
**Three Factors Model** POQ and CI combined	1203 (227)	730 (3***)	5.30	.82	.84	.09
**Three Factors Model** POQ and CD combined	1463 (227)	990 (3***)	6.45	.77	.79	.10
**Two Factors Model** POQ, CD, and ANX combined	2396 (229)	1923 (5***)	10.46	.60	.64	.13
**Single Factor Model**	2957 (230)	2484 (6***)	12.86	.50	.55	.15
**Study 2**						
**Five Factors Model**	776 (289)	-	2.69	.92	.93	.06
**Four Factors Model** POQ and LS combined	1203 (293)	427 (4***)	4.11	.86	.87	.08
**Four Factors Model** POQ and CI combined	1397 (293)	621 (4***)	4.77	.83	.84	.09
**Three Factors Model** POQ, CI and LS combined	1808 (296)	1032 (7***)	6.11	.76	.78	.10
**Two Factor Model **POQ, CI, LS and CD combined	2388 (298)	1612 (9***)	8.01	.67	.70	.12
**Two Factor Model **POQ, CI, LS and ANX combined	2560 (298)	1784 (9***)	8.59	.65	.68	.12
**Single Factor Model**	3148 (299)	2372 (10***)	10.53	.56	.59	.14

*POQ* Overqualification, *CI* Career Identity, *CD* STEM Career Decidedness, *ANX* STEM Career Anxiety, *LS* Leader Support

### Hypothesis testing

SEM was used to test our proposed hypotheses, and the results are exhibited in Table 3. Perception of STEM-based overqualification had a positive link with STEM-based CI (β = 0.23, *p* < .01), supporting H1. In addition, STEM-based CI had a negative association with STEM-based CA (β = − 0.20, *p* < .01), supporting H2a. Finally, STEM-based career identity had a positive relationship with STEM-based CD (β = 0.45, *p* < .001), supporting H3a.

**Table 3:** Study 2 results of direct and moderation hypotheses

Path	Study 1 coefficients (β)	Study 2 coefficients (β)	Supported
**H1**: POQ –> CI	0.23^**^	0.30^***^	Yes
**H2a**: CI –> ANX	− 0.20^**^	− 0.19^**^	Yes
**H3a**: CI –> CD	0.45^***^	0.34^***^	Yes
**Study 2 Moderation analysis**			
POQ*LS–> Career Identity		0.20^***^	Yes

*POQ* Overqualification, *CI* Career Identity, *CD* STEM Career Decidedness, *ANX* STEM Career Anxiety, *LS* Leader Support

**p < .01

***p < .001

### Indirect effect test

In this study, we employed the bootstrap approach (bootstrapping sample size = 5,000) to examine the indirect role of STEM-based CI in the link between POQ and STEM-based CA and between POQ and STEM-based CD. Table 4 indicates the results for the intermediary mechanisms. These results indicated that STEM-based CI mediated the link between POQ and STEM-based CA (indirect effects = − 0.05, LL = − 0.07, UL = − 0.03), because the CIs did not include 0 (Table 4). Therefore, H2b was supported. Similarly, CI mediated the link between POQ and CD (indirect effects = 0.10, LL = 0.07, UL = 0.15), because the CIs did not contain zero, thus supporting H3b.

**Table 4:** Study 1 and study 2 mediating effects of POQ through CI on outcome variables

Study 1	STEM Career Anxiety	C.I. limits	STEM Career Decidedness	C.I. limits
Career Identity	− 0.05	− 0.07, − 0.03	0.10	0.07, 0.15
**Study 2**				
Career Identity	− 0.057	− 0.09, − 0.04	0.10	0.07, 0.14

CI = Confidence Interval limit 95%; Bootstrap sample size = 5000; IV = Overqualification, MV = Career Identity, DV = STEM Career Anxiety and STEM Career Decidedness

#### Study 2

Study 2 was conducted to overcome the Study’s 1limitation. First, as Study 1 was cross-sectional and the data were collected from a single source at one point in time, the results may have lacked authenticity. To overcome this issue, in Study 2, we collected data at three time points. Second, the respondents in Study 1 were all faculty members from schools, colleges, and other teaching institutions, which limited the generalizability of the results. Therefore, in Study 2, we collected data from engineering firms’ women employees (from an industry perspective) to confirm the findings of Study 1 in a different setting. Finally, we tested the effect of leader support as a boundary condition; specifically, we tested whether leader support affects the hypothesized relationships directly (direct moderating effect) or indirectly (moderated mediation).

## Method

In Study 2, we utilized a time-lag design to collect data from the respondents in three waves to reduce CMB. We also analyzed the data using moderated mediation analysis. Our goal was to examine the moderating role of LS on STEM employees’ CI and the moderating role of LS on the mediator (CI), to identify the relationship between STEM employees’ POQ and their CA and CD. We used a two-month gap between the data collection stages. Initially, with the help of their respective HR departments, we invited 950 early-career women from the participating firms. At Time 1, we collected the female employees’ demographic information and POQ and received 692 valid responses. Two months after Time 1, we sent the CI and LS measures to these 692 respondents and received 588 complete responses. At Time 3, two months after Time 2, we sent the CA and readiness measures to the respondents who had completed the survey at Time 2. We received 515 valid responses from early career engineers working in construction, design, and electrical firms.

### Measures

We used a 3-item scale of leader support scale developed by [4]. On this scale, we asked early-career engineers to rate their immediate supervisors (leader support). Item included “*My supervisor encourages and supports me when I have a difficult and stressful innovation task or responsibility.*” In addition, we used the same measurement scales as in Study 1 to measure POQ, CI, CA, and CD. We also controlled for the same demographic variables as in Study 1 and Table 1 provides detailed demographic statistics.

## Data analysis

### CFA

In the current research, we employed AMOS to conduct CFA, validating the suggested study variables (refer to Table 2). Specifically, we first merged the items with the highest and lowest factor loadings for each construct based on factor analysis results, then assigned all items to an indicator. The CFA model tested in Study 2 was a five-factor model: perception of STEM-based overqualification, STEM-based CI, STEM-based CA, STEM-based CD, and LS to evaluate the fit of the model, TLI, CFI, and RMSEA were measured. As displayed in Table 2, the results revealed an acceptable model fit: χ^2^/df = 2.69, *p* ≤ .01, CFI = 0.92, TLI = 0.93, RMSEA = 0.06. The findings revealed that all factor loadings significantly influenced the latent constructs once convergent validity was confirmed. Fit indices demonstrated that the five-factor model provided a better fit for the data compared to other models (refer to Table 2). Consequently, the core construct outcomes of the study are more distinctive and supportive of the results. Furthermore, as in Study 1, the first three hypotheses were supported (see Tables 3 and 4).

### Hypothesis testing

#### Moderating analysis

**Fig. 2:**
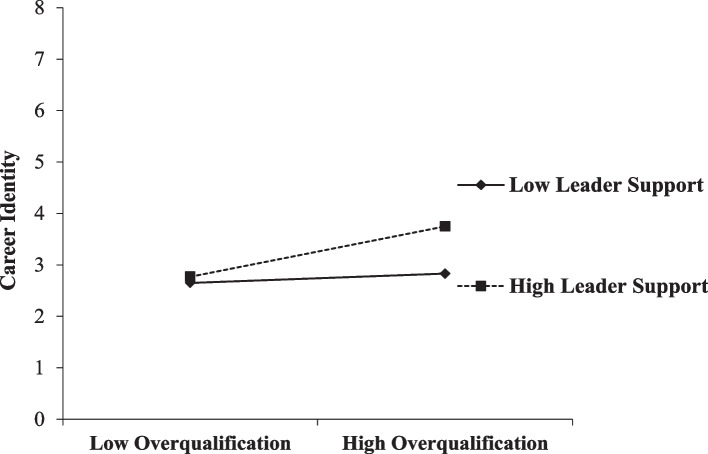
Moderating role of Leader Support on the Overqualification- Career Identity Relationship (Study 2)

Table 3 shows the moderating role of LS on the association between the perception of STEM-based overqualification and STEM-based CI. The findings indicate that the interaction between perceptions of STEM-based overqualification and LS had a significant effect on CI (β = 0.20, *p* < .01). Therefore, H4 was supported. We plotted the interactive effect to determine the moderating influence of LS by using the [52] method (see Fig. 2). Figure 2 indicates that the positive relationship between the perception of STEM-based overqualification and CI (*r* = .49, *p* < .001) was stronger when the level of LS was high than when it was low (*r* = .09, *p* > .05).

#### Moderated mediation analysis

Table 5 exhibits the CIs in the bootstrap test for the indirect effects of leader support on STEM-based CA through STEM-based CI. The bootstrapping CIs for the indirect LS values at one SD above the mean were (-0.15 to − 0.06) and did not contain 0, indicating significant indirect effects, which was confirmed by the significant index of moderated mediation is also significantly reported in Table 5. Therefore, H5a was supported. Table 5 also indicates the bootstrapping CIs for the indirect effect on STEM-based CD for leader support values are one SD above (0.12 to 0.24), mean (0.07 to 0.14), and one SD below the mean (-0.01 to 0.06). Since at one SD above and at mean level does not contain 0, indicating the significant moderated mediated effect of POQ on CD through STEM-based CI under the moderating effect of LS. In addition, the index of moderated mediation was statistically significant (see Table 5), supporting H5b.

**Table 5:** Study 2 conditional indirect effects of overqualification through career identity on STEM career anxiety and STEM career decidedness at the levels of Leader Support

Leader Support	Boot indirect effects	Boot SE	Boot Lower limit 95% CI	Boot Upper limit 95% CI
**Indirect effects on STEM Career Anxiety**				
-1 SD	− 0.01	0.01	− 0.03	0.01
Mean	− 0.06	0.01	− 0.09	− 0.04
+ 1 SD	− 0.10	0.02	− 0.15	− 0.06
***Index of Moderated Mediation***				
	− 0.04	0.01	− 0.06	− 0.02
**Indirect effects on STEM Career Decidedness**				
-1 SD	0.02	0.02	− 0.01	0.06
Mean	0.10	0.02	0.07	0.14
+ 1 SD	0.17	0.03	0.12	0.24
***Index of Moderated Mediation***				
	0.07	0.01	0.04	0.10

## Discussion

Women employees’ perceptions of their qualifications and their impact on themselves and their organizations have received increasing attention from researchers. The impact of perceived overqualification on employees’ satisfaction, job attitude, task success, extra-role behaviors, and irregular behavior has also been studied [18, 20]. However, the effect of POQ on the professional achievements of employees has received little attention. In this study, we developed a model to investigate how STEM women’s POQ influences their CI, in turn influencing their CA and CD, using RIT and the symbolic interactionist perspective of role identification. We also examined the strength of the positive link between POQ and CI in the context of supportive leadership. By confirming all our hypotheses, our research makes a significant contribution to the literature on overqualification among STEM employees. Our findings also add to the growing body of knowledge in HR management, especially in leadership, career management, and organizational behavior.

### Theoretical implications

Few studies have been done on the impact of POQ on STEM women’s career outcomes. This research fills this gap in the literature by showing that STEM women’s POQ is related to a strong CI. Specifically, we investigated how STEM women’s behaviors and attitudes in the workplace are influenced by their POQ. Through the mediating effect of CI, our results also showed that POQ is indirectly related to a reduction in CA and an increase in CD.

Our findings support RIT in research on overqualification in the STEM context. Several theoretical models have been used to describe the impact of overqualification, including the person-job fit theory and relative deprivation theory [53]. Studies have explored how workers’ perceived overqualification affects their career-related behavioral outcomes [18]. Yet, there have been few studies on how employees’ self-perception, career-related habits, and attitudes are affected by their POQ in STEM fields. We demonstrate that POQ can influence the career-related outcomes of early career employees after extending role identity theory to the STEM context. Our results also show that career identity plays a key role in the relationship between employees’ perceived overqualification and their professional achievements. Managing STEM workers who feel overqualified expect a thorough understanding of their needs. Our findings suggest that employees’ POQ can strengthen their CI and that professionals who experience overqualified might have a greater need to make clear CD.

Furthermore, we showed that STEM women who feel overqualified are more prone to establish a strong CI with the aid of supportive leaders, helping them to validate their positive self-view. Overqualification in STEM fields has been shown to be detrimental in many studies. Recent research has also examined how workers who feel overqualified may make positive use of their overqualification (Ma et al., 2020). According to the results of this study, POQ can strengthen STEM women’s CI under supportive leadership. Our findings add to the growing body of knowledge of the positive outcomes of STEM women’s overqualification [2]. Moreover, research on perceived overqualification in STEM fields has shown that self-efficacy is the primary mechanism by which perceived overqualification influences employee performance [5, 54].

Ignoring the effect of self-efficacy in this study, we found that STEM CI is another important mechanism by which STEM employees’ POQ affects their career outcomes. In addition to addressing social similarities, people’s unsuitability, and psychological deprivation for career development, we investigated the effect of POQ on the career outcomes of STEM employees early in their careers. Although numerous boundary conditions can be used to shape the perceived effect of STEM overqualification, few empirical studies have examined the moderators of POQ. For example, researchers have defined and debated the roles of various moderating variables, such as peer overqualification [12]. However, up to this point, leader support has not been examined as a moderating factor influencing the effect of POQ on STEM employees’ career outcomes, despite leadership being a critical workplace context factor. For instance, leadership has been proven to significantly impact employees’ attitudes and behaviors. In this research, we considered the symbolic interactionist perspective of role identity and examined leader support as a moderating factor that enhances the positive influence of POQ on STEM employees’ CI. In addition, contextual factors have been studied to determine whether they can mitigate the negative effect of overqualification on employee behavior (Simon et al., 2019). In this study, we took a unique approach to demonstrate how contextual variables can enhance the positive impact of POQ on the behaviors and attitudes of early-career STEM professionals.

### Managerial implications

This study provides several managerial implications. Firstly, the positive relationship between STEM-based overqualification perception and STEM-based CI recommends that businesses acknowledge and reward the credentials and talents that women bring to STEM professions. Managers can create an environment in which women’s overqualification is viewed as a benefit rather than a disadvantage. This can be accomplished by giving women an opportunity to exhibit their ability, assigning them difficult projects, and including them in decision-making processes. Organizations can improve job happiness and motivation among overqualified women by recognizing and embracing their STEM-based career identity. Second, the negative relationship between STEM-based CI and STEM-based CA emphasizes the significance of providing support and tools to help women overcome career anxiety. Managers should establish mentorship programs, aid, and counseling, and build a supporting network for women in STEM fields. Organizations can encourage a sense of confidence and well-being among overqualified women by resolving professional anxiety, allowing them to pursue their career goals with greater clarity and drive. Third, the positive relationship between STEM-based career identity and STEM-based CD highlights the importance of businesses facilitating career development options for overqualified women. Managers should offer training programs, skill-building initiatives, and advancement opportunities that fit with women’s objectives and skills in STEM professions. Organizations may foster the professional progress and long-term commitment of overqualified women by investing in their career determination, resulting in higher retention and satisfaction.

Fourth, our findings highlight the importance of leadership as a management trait, which is particularly useful in recruiting and deploying female staff who believe that their STEM skills are scarce for their role. HR management is incomplete without supportive leadership [55], just as organizational strategies may need to be adjusted to meet the specific needs of overqualified early-career women in STEM fields. Our findings imply that leaders can modify their leadership practices to optimize the advantages gained from overqualified employees. Given the moderating role of LS, our results indicate that even if managers cannot adjust their employees’ roles, they can enhance their comprehension of STEM-based qualifications by providing support. This finding supports the notion that businesses should strive to foster a positive contact environment between workers and their company [20, 56]. Our findings also indicate that managers can use the support of leaders to develop better teams. Indeed, as more and more workers report feeling overqualified at work, leader support will become an increasingly important management behavior in the future. Organizations should realize the importance of such support and offer basic skills training for leaders to help them guide and support their employees.

Finally, our research provides insight into and suggestions for the recruitment, selection, and development of early-career employees in light of the potential value of STEM overqualification. STEM overqualification should not be understood as solely negative. Although early career employees have more experience than unqualified workers, hiring them can be an economical way to recruit highly experienced workers. According to our findings, early career STEM professionals who feel overqualified have a strong CI and are more prone to have less CA and to make better career decisions. These positive effects of overqualification are more pronounced when leaders are involved. Although recruiting highly skilled early-career individuals can present some risks [20], careful management of these hiring decisions will translate into benefits. Therefore, when making hiring decisions, HR experts should not necessarily choose applicants who think their STEM skills are too high.

### Limitations

Some limitations of this study should be considered. First, we examined STEM overqualification from the viewpoint of early career employees, which has been based on a combination of employee- and supervisor-rated overqualification perceptions [18, 35]. We designed our research to focus on STEM employees’ life observations and how they influence their CI and career outcomes. Future studies possibly investigate the effects of objective overqualification in STEM or other specialist disciplines using career entry criteria and expert ratings of employee POQ.

Second, other types of leadership behaviors or leader variables, in addition to leader support, may contribute to positive social interactions at work. When leader–employee relationships are positive [57], early career STEM employees may use positive cues from these relationships when evaluating their own career identity. Other leadership styles could be examined in future studies to replicate our results. This type of investigation could provide interesting information on leaders’ personalities and leadership styles, helping them to help manage early-career employees who feel overqualified.

Third, our findings reveal a link between POQ and CA through CI. Researchers have found that POQ among early career professionals in STEM fields is linked to psychological outcomes and lower work satisfaction [48]. As a result, an important extension of our research would be to examine the underlying mediation and moderation mechanisms involved in improving employees’ subjective well-being by reducing the negative effects of POQ. CA, for example, can be viewed as a short-term experience despite its long-term focus, and CD can also be a short-term state. As a result, this study focused on short-term career-related behaviors and attitudes. When investigating the impact of POQ on STEM employees’ attitudes and behaviors, future research should consider time orientation.

Fourth, the data used in our two studies came from two different industries (education and engineering), although both studies were conducted in the same cultural context (i.e., China). As we used samples from China, which has a strong collectivist culture, our findings may be influenced by unique cultural characteristics [58]. Therefore, management researchers could use data sets from different cultures and industries to expand and test our results in future studies.

Finally, a flaw common to all applicable data is the inability to establish causality. Like previous studies using a similar design, our research is limited by this restriction [5]. Early career STEM professionals who actively choose their careers can develop a strong CI, according to our hypothetical model. As we obtained information from a single source (early career employees) but from different sectors (education and engineering), our hypothetical model helped to alleviate reverse causality issues; in addition, in Study 2, we used a time-lag approach. Nevertheless, to further reduce the impact of CMB and social desirability, verify causality, and rule out other possibilities, it would be useful to test our research model using a longitudinal approach.

## Conclusion

In this study, we investigated how early career STEM employees’ POQ influences their CI, in turn affecting their CA and CD. Our results suggest that the career growth of STEM employees can be facilitated by their POQ, which strengthens their CI, trims down their career uncertainty, and encourages them to make career decisions. We also show that when leaders display encouraging attitudes, the beneficial effect of POQ on early-career STEM employees’ CI is stronger. Specifically, our results reveal that POQ can contribute to positive career development outcomes for STEM employees, especially when the level of leader support is high.

## Data Availability

Data for this study can be attained at the request from the corresponding author.* Corresponding author. Ali Ahmad Bodla E-mail address: ali.ahmad@ru.nl
